# Assessment of Complete Plastid Genome Sequences of *Tulipa alberti* Regel and *Tulipa greigii* Regel Species from Kazakhstan

**DOI:** 10.3390/genes15111447

**Published:** 2024-11-09

**Authors:** Shyryn Almerekova, Moldir Yermagambetova, Anna Ivashchenko, Saule Abugalieva, Yerlan Turuspekov

**Affiliations:** 1 Institute of Plant Biology and Biotechnology, Almaty 050040, Kazakhstan; almerekovakz@gmail.com (S.A.); ermaganbetova.moldir@bk.ru (M.Y.); absaule@yahoo.com (S.A.); 2 Institute of Zoology, Almaty 050060, Kazakhstan

**Keywords:** *Tulipa*, plastid genome, protein-coding genes, DNA barcoding, phylogenetic analysis

## Abstract

Background. *Tulipa* species are economically, culturally, scientifically, and ecologically important. Tulips present taxonomic complexities that cannot be adequately resolved by examining their morphological characteristics alone or by relying on a limited selection of genetic markers. Methods. In the present study, we assessed the complete plastid sequences of *Tulipa alberti* Regel and *Tulipa greigii* Regel collected from Kazakhstan. Additionally, 14 previously published plastomes were obtained from GenBank for comparison and phylogenetic analysis. Results. The plastid genome sizes of *T. alberti* and *T. greigii* were 152,359 bp and 152,242 bp, respectively. In the plastid genomes of *T. alberti* and *T. greigii*, 136 genes were annotated, 114 of which were unique. These unique genes comprised eighty protein-coding, thirty transfer RNA, and four ribosomal RNA genes. Additionally, 415 simple sequence repeats were identified, comprising 107 tandem, 40 forward, 49 palindromic, 8 reverse, and 1 complementary repeat. Notably, the region containing *ycf1* exhibited high variability and may serve as an informative DNA barcode for this genus. Conclusion. Phylogenetic analysis showed strong support for the relationships among *Tulipa* species, indicating the utility of plastid genome data for further taxonomic studies within the genus.

## 1. Introduction

*Tulipa* L. is an important genus within the family Liliaceae Juss [1]. The *Tulipa* system classifies species into four subgenera and twelve sections, mainly based on nuclear genome sizes and morphological data [1,2]. The genus comprises ca. 150 wild species [2,3,4,5,6], including up to 55 species in the subgenus *Tulipa* [2,5]. The subgenus *Tulipa* comprises seven sections, including the *Kolpakowskianae* Raamsd. ex Zonn. & Veldk. (16 species), *Multiflorae* (Raamsd.) Zonn. (3), *Lanatae* (Raamsd.) Zonn. (7), *Vinistriatae* (Raamsd.) Zonn. (6), *Spiranthera* Vved. ex Zonn. & Veldk. (4), *Tulipanum* Reboul (10), and *Tulipa* (9) sections [2].

Classification has been updated by utilizing plastid regions, resulting in enhanced analysis [5]. With the advancement of molecular genetic methods, numerous studies utilizing Sanger sequencing have been conducted [7,8,9,10], particularly focusing on the internal transcribed spacer region as well as the plastid regions. All the studies mentioned above have contributed significantly to establishing and evaluating evolutionary relationships within the *Tulipa* genus. Nevertheless, the precise taxonomy of *Tulipa* remains poorly understood because of the close morphological similarities among species, hybridization, synonyms, and the emergence of numerous new species [5].

The Tian Shan and Pamir-Alay mountains are considered one of the principal regions for the origin of *Tulipa* species [2,11,12], and contain approximately 80 species [13]. Particularly, in Kazakhstan, there are 42 species of *Tulipa* [14], 18 of which are endemic, rare, and endangered species [15]. Two of these rare species, *T. alberti* Regel and *T. greigii* Regel, are included in the weakly studied section *Vinistriatae* [2] and were analyzed in this study using plastid genomes. The section *Vinistriatae* includes six species: *Tulipa micheliana*, *Tulipa vvedenskyi*, *Tulipa mogoltavica*, *Tulipa butkovii*, *Tulipa albertii*, and *Tulipa greigii* [2]. However, a clear separation of species from this section within the subgenus *Tulipa* is uncertain [5].

Plastids, which are important organelles found within plant cells, play a vital role in photosynthesis and possess unique genomes [16]. Typically, the plastid genome exhibits a structure characterized by circular DNA molecules [17,18] and a quadripartite structure [19,20]. Plastid genomes are conserved in organization and structure and contain a relatively conserved set of genes [21,22].

Conserved genome structure and stable gene composition of plastid genomes serve as valuable tools for phylogenomic analysis [23,24] and function as superbarcodes [25,26]. Additionally, specific regions within the plastid genomes have been used for species identification [27,28] and DNA barcoding [29,30]. With rapid advancements in next-generation sequencing technology, obtaining plastid genome sequences has become increasingly efficient and accessible. Plastid genomes contain numerous informative regions, including highly variable intergenic spacers and protein-coding sequences, which enable the resolution of phylogenetic relationships at various taxonomic levels [31]. Additionally, the incorporation of plastid genome sequences into phylogenetic analyses offers a powerful and cost-effective approach for elucidating evolutionary relationships and understanding the diversity and evolution of plant species [32].

The sequencing of plastid genomes within the *Tulipa* genus began in 2019 with the publication of the genome of *T. altaica* [33]. Since then, the complete plastid genomes of several other species have also been reported [34,35,36,37,38]. The first comparative analysis of five *Tulipa* plastomes was conducted by Li et al. [39]. However, despite the existence of sequenced *Tulipa* plastomes, complete sequences of species belonging to the section *Vinistriatae* have not been reported. The findings from the plastid genome analysis described in this section will provide valuable data for further taxonomic studies of *Tulipa*. The expansion of plastid genome sequencing in Tulipa is important for molecular systematics study in the genus, the search for new informative markers, the evaluation of genetic variability in populations, and the development of conservation strategies for endangered species of the genus.

This study aimed to assess the complete plastid genomes of *T. alberti* and *T. greigii*, two species within the section *Vinistriatae* (subgenus *Tulipa*), using next-generation sequencing technology. This study focused on (1) the assessment of the plastid genome organizations in two *Tulipa* species in the section *Vinistriatae*, (2) the conduction of a comparative analysis by integrating the plastid genomes of other species of the genus available in GenBank, (3) the identification of polymorphic regions within *Tulipa* plastomes suitable for DNA barcoding purposes, and (4) the evaluation of the phylogenetic placement of *T. alberti* and *T. greigii* the relied on the alignments of complete plastid genome sequences.

## 2. Materials and Methods

### 2.1. Leaf Materials and DNA Isolation

Plant materials of *T. alberti* and *T. greigii* were collected from the Almaty (Malaysary pass, right bank of the Ili River) and Zhambyl (Kordai pass, right side of the road) regions of Kazakhstan, respectively, and dried in silica gel for further DNA isolation. Tulip plant materials were sampled from generative individuals without harming natural populations, with permission from the Committee of Forestry and Wildlife of the Ministry of Ecology, Geology, and Natural Resources of the Republic of Kazakhstan. Dried leaves of *Tulipa* species were used for DNA isolation using the cetyltrimethylammonium bromide protocol [40].

### 2.2. Genome Sequencing, Assembly, and Annotation

The sequencing component of this study followed the methodology outlined in our previous studies, which focused on characterizing the plastid genomes within *Juniperus* species [41,42,43]. DNA samples from *Tulipa* species were used for library development using a TruSeq Nano DNA Kit (Illumina Inc., San Diego, CA, USA). Plastome sequencing of *T. alberti* and *T. greigii* was performed using an Illumina NovaSeq 6000 platform (Illumina Inc., USA) at Macrogen Inc. (Seoul, Republic of Korea). The quality control assessments of the obtained nucleotide sequences were conducted based on FastQC (http://www.bioinformatics.babraham.ac.uk/projects/fastqc, accessed on 21 February 2024). The obtained sequencing data were analyzed using Trimmomatic [44] to remove the adapter sequences. NOVOPlasty 4.3.3 [45] was used to assemble and obtain clean reads, with *T. altaica* (MW077741.1) used as a reference. The Ge Seq [46] was applied to assemble plastid genome nucleotide sequences of *T. alberti* and *T. greigii*. The sequences were manually corrected using the reference genome (MW077741.1). Circular plastid genome maps for *T. alberti* and *T. greigii* were generated using Organellar Genome DRAW 1.3.1 (OGDRAW) [47]. The annotated plastome nucleotide sequences of *T. alberti* and *T. greigii* were deposited in the National Center for Biotechnology Information (NCBI) GenBank.

### 2.3. Analysis of Repeat Sequences

The simple sequence repeat (SSR) position and types were selected utilizing the MISA software v2.1 (https://webblast.ipk-gatersleben.de/misa/, accessed on 23 August 2024) [48]. Thresholds were set for mononucleotide SSRs at eight repeats; for dinucleotides and trinucleotides at four repeats; and for tetranucleotides, pentanucleotides, and hexanucleotides at three repeats. The REPuter tool (https://bibiserv.cebitec.uni-bielefeld.de/reputer, accessed on 26 August 2024) [49] was used to detect forward (F), reverse (R), and palindromic (P) repeat elements, utilizing the parameter settings of Hamming distance = 3 and a minimum repeat size of 30 bp. Tandem repeat sequences were identified using the default settings of the tandem Repeats Finder program [50].

### 2.4. Plastome Analysis by Sliding Window, IR Regions Contraction, and Expansion

Nucleotide diversity (Pi) of the complete plastid genome of *Tulipa* species was calculated using the DnaSP version 6.0 (DNA Sequence Polymorphism) package [51] with a step size of 200 bp and a window length of 600 bp. The complete plastid genome sequences of the *Tulipa* species were aligned using MUSCLE [52] in Geneious Prime^®^ 2024.0.2 (https://www.geneious.com, accessed on 26 August 2024). The junction sites of *Tulipa* plastomes were examined and visualized using the online tool IRscope (https://irscope.shinyapps.io/irapp/, accessed on 16 May 2024) [53].

### 2.5. Analysis of Phylogenetic Relationships

Phylogenetic analysis was performed using the alignment of complete plastid genome sequences, common protein-coding gene sequences, and *ycf1* gene sequences from *T. alberti* and *T. greigii* species collected in Kazakhstan from this study, 12 *Tulipa* samples obtained from NCBI GenBank, and 2 outgroup samples, *Amana edulis* (OL351568) and *Erythronium japonicum* (MT261155). The nucleotide sequences of *Tulipa* and outgroup species were aligned using Geneious Prime^®^ 2024.0.2 (https://www.geneious.com, accessed on 26 August 2024). Phylogenetic analyses were performed using the maximum likelihood (ML) method. The best-fitting model for all alignments was selected using multiple tests in IQ-TREE version 2.2.2.6, employing both the Akaike Information Criterion (AIC) and Bayesian Information Criterion (BIC). According to the BIC, the best-fitting models were TVM+F+I+R5, K3Pu+F+I, and TVM+F+I, and according to the AIC, the best-fitting models were TVM+F+I+R5, K3Pu+F+I+G4, and GTR+F+I for the complete plastid genome, common protein-coding genes, and *ycf1* gene sequences, respectively. The ML phylogenetic tree was then reconstructed using the nucleotide substitution models selected based on the BIC via IQ-TREE version 2.2.2.6 software [54], as BIC reduces the risk of overfitting and enhances the accuracy of phylogenetic analyses [55]. The generated trees were visualized using FigTree (http://tree.bio.ed.ac.uk/software/figtree/, accessed on 3 September 2024). The subgenera and section names of the samples used in this analysis followed the taxonomy outlined by Veldkamp and Zonneveld [1].

## 3. Results

### 3.1. Tulipa Plastid Genome Features

**Figure 1:**
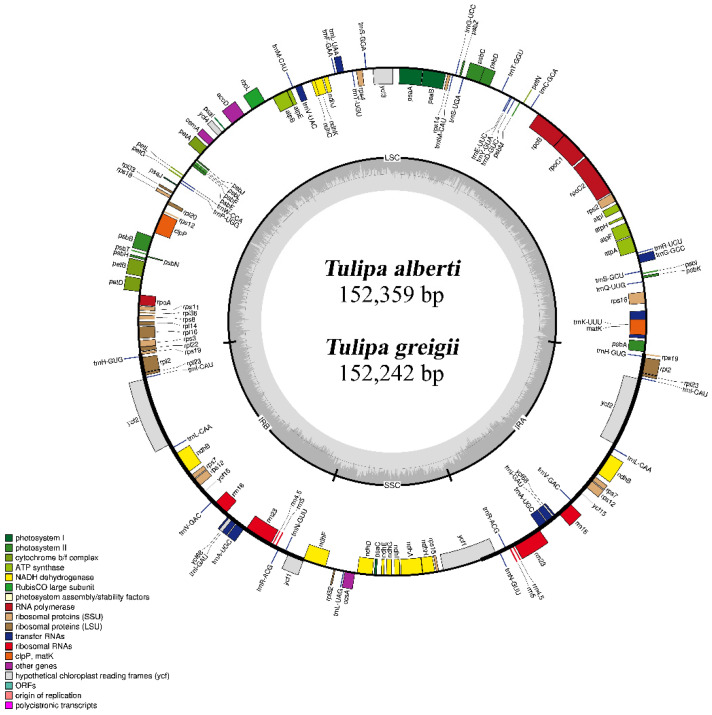
The plastid genome map of *Tulipa alberti* and *Tulipa greigii* generated using OGDRAW 1.3.1 software. In the visualization, darker and lighter grey shades represent regions with higher GC and AT contents, respectively. The plastid genome is segmented into the large single-copy region (LSC), small single-copy region (SSC), inverted repeat region A (IRa), and inverted repeat region B (IRb) regions, with genes from different functional groups color-coded accordingly.

The plastid genomes of *T. alberti* and *T. greigii* contained a singular circular molecule with a typical quadripartite structure. The *Tulipa* plastome consists of two IR regions and two single-copy regions, including an LSC and SSC (Figure 1).

The sizes of the *T. alberti and T. greigii* plastomes were 152,359 and 152,242 bp, respectively. The lengths of the IR regions were 52,744 and 52,742 bp, with a GC content of 42.01% in both cases. The GC content in the LSC and SSC regions was 34.49% for *T. alberti,* 35.53% for *T. greigii*, 29.85% for *T. alberti,* and 29.88% for *T. greigii*. The total GC content in the studied samples was 36.57% and 36.59% in the *T. alberti* and *T. greigii* plastomes, respectively. The complete plastome sequences of *T. alberti* and *T. greigii* were deposited in GenBank under the accession numbers OR456441 and PP338772, respectively (Table 1).

In total, 136 genes were annotated in the plastomes of *T. alberti* and *T. greigii*, of which 114 were unique. Among these, eighty were protein-coding, thirty were transfer RNA (tRNA), and four were ribosomal RNA (rRNA) genes. Eighteen intron-containing genes were identified in the *T. alberti* and *T. greigii* plastomes. These intron-containing genes included twelve protein-coding and six tRNA genes (Table 2). The majority of the genes were located in the LSC and SSC regions, with 22 of 136 genes in the IR regions being duplicated. These duplicated genes included ten protein-coding, eight tRNA, and four rRNA genes (Table 2).

### 3.2. Repeat Elements Analysis

**Figure 2:**
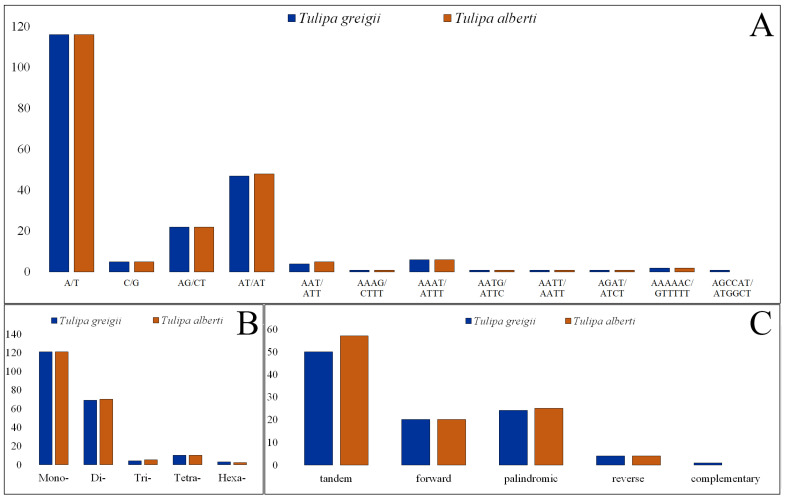
Analysis of repeat sequences of *T. greigii* and *T. alberti* plastomes. (**A**) Frequency of repeats with different motifs; (**B**) number of different sample sequence repeat types; (**C**) number of specific long repeats.

Using MISA, 207 and 208 SSRs were identified in the plastid genomes of *T. greigii* and *T. alberti*, respectively. Most of the SSRs (242; 58.31%) in the studied plastomes featured a mononucleotide repeat motif. The second most prevalent repeat motif was dinucleotide SSRs, which comprised 139, or 33.49%, of the total SSRs. Nine (2.17%) trinucleotide, twenty (4.82%) tetranucleotide, and five (1.20%) pentanucleotide SSRs were detected in the two analyzed *Tulipa* plastomes (Table 3; Figure 2A). Most SSRs consisted of A/T (232) and AT/AT (95) motifs in the mononucleotide and dinucleotide repeats, respectively (Table 3; Figure 2B). The majority of the detected SSRs were found in the intergenic and LSC regions of *Tulipa* plastid genomes (Supplementary Table S1).

We then analyzed tandem, forward, palindromic, reverse, and complement repeat elements in the two *Tulipa* samples. In total, 107 tandem repeats, 40 forward repeats, 49 palindromic repeats, 8 reverse repeats, and 1 complementary repeat were observed. Complementary repeats were exclusively identified within the *T. greigii* plastome (Figure 2C).

### 3.3. Sliding Window Analysis

**Figure 3:**
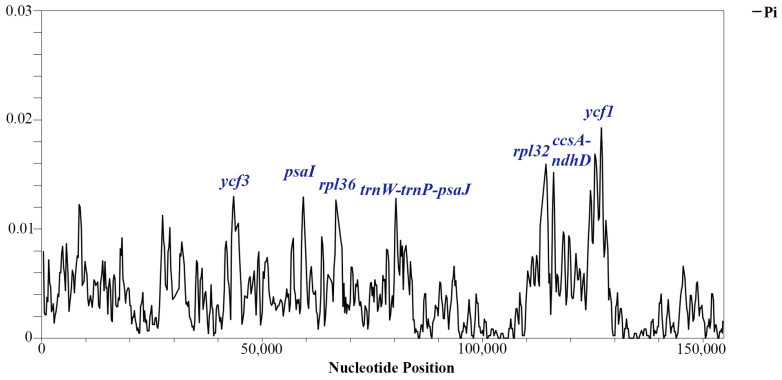
Nucleotide variability (Pi) analysis in complete plastid genome sequences of *Tulipa* plastomes. The labels at the peaks indicate the gene regions with the highest Pi values.

Across the analyzed protein-coding genes, the Pi values ranged from 0 to 0.01927, averaging with 0.003873. Notably, *ycf1* exhibited the highest variation (Pi value—0.01927). Additionally, multiple variable hotspots were detected. Furthermore, seven regions displaying relatively high levels of variability were identified in the analyzed *Tulipa* protein-coding genes. These regions, including *trnW-trnP-psaJ*, *rpl36*, *psaI*, *ycf3*, *ccsA-ndhD*, *rpl32*, and *ycf1*, exhibited Pi values of ≥ 0.01 (Figure 3).

### 3.4. IR Region Contraction and Expansion

**Figure 4:**
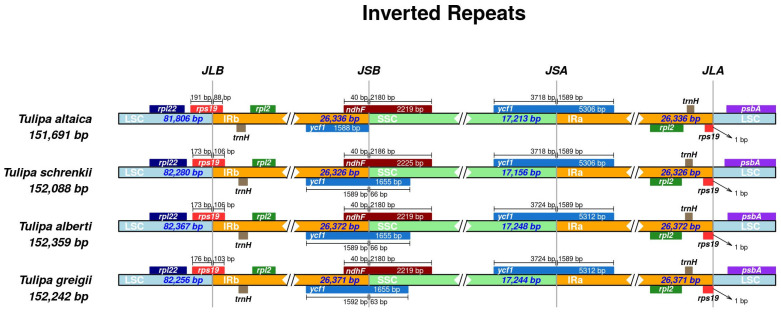
Comparisons of the borders of a large single-copy (LSC), inverted repeat (IR), and small single-copy (SSC) regions among *Tulipa* plastid genomes. JLB (LSC/IRb), JSB (IRb/SSC), JSA (SSC/IRa), and JLA (IRa/LSC) denote the junction sites between the two corresponding regions in the four plastomes.

Using IRscope, contraction and expansion in the junction regions of the large single-copy, small single-copy, and inverted repeat were examined. This analysis encompassed plastomes from *T. alberti* and *T. greigii* species belonging to the section *Vinistriatae*, as investigated in this study, along with *T. schrenkii* (OL350836) from the section *Tulipa* and *T. altaica* (MW077741) from the section *Kolpakowskianae*, which were sourced from GenBank. Across all four compared *Tulipa* plastomes, *rpl22* and *psbA* were consistently situated within the LSC region, whereas *rpl2* and *trnH* were entirely contained within the IR regions. The location of the *rps19* found at the inverted repeat A/large single-copy and large single-copy/inverted repeat B junctions, with the integration of 106 bp into the inverted repeat B in *T. schrenkii*, *T. alberti*, and *T. greigii*. Furthermore, *ndhF* and *ycf1* extended beyond the inverted repeat B/small single-copy junctions, with 40 and 1589 bp (*T. schrenkii* and *T. alberti*) and 1592 bp (*T. greigii*) included in the inverted repeat B region, respectively. The copy of the *ycf1* was also observed at the inverted repeat A/small single-copy junction in all four *Tulipa* plastid genomes, which was incorporated into the inverted repeat A by 1589 bp. The inverted repeat A, inverted repeat B, and small single-copy regions in *T. alberti* and *T. greigii* were slightly longer than those in the two non-*Vinistriatae* species (Figure 4).

### 3.5. Phylogenetic Analysis

**Figure 5:**
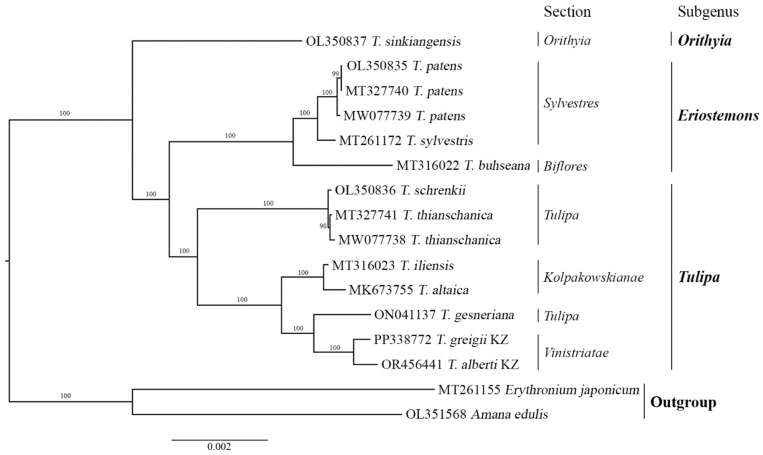
Maximum likelihood dendrogram of *Tulipa* species using nucleotide sequences of the plastid genome. The letters “KZ” represent species collected from Kazakhstan.

ML assessment was conducted based on complete plastid genome sequences from 16 samples. The total aligned length of the complete plastid genome sequence was 156,094 bp. The resulting phylogenetic tree delineated three primary clades corresponding to three subgenera (*Orithyia*, *Eriostemones*, and *Tulipa)*. Notably, the specimens of *T. alberti* and *T. greigii* collected from Kazakhstan together with *T. gesneriana* (section *Tulipa*) formed a distinct subclade within the *Tulipa* subgenus clade (Figure 5).

In addition, the ML-based tree was reconstructed using nucleotide sequences of protein-coding genes (Supplementary Figure S1). The phylogenetic trees based on the sequences of the whole plastomes (Figure 5) and protein-coding genes exhibited similar topologies (Supplementary Figure S1).

**Figure 6:**
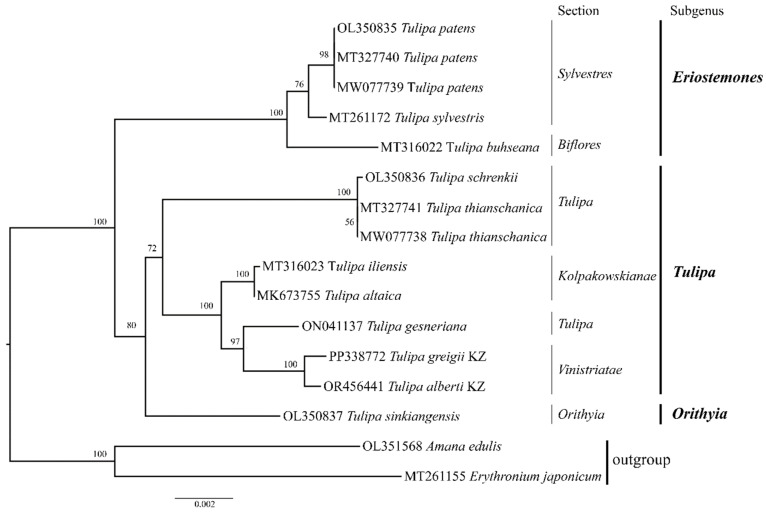
Maximum likelihood tree of *Tulipa* species using nucleotide sequences of the *ycf1*. The letters “KZ” represent species collected from Kazakhstan.

The region of the *ycf1* gene showed the highest polymorphism in the examined protein-coding genes (Figure 3). The sequences of the *ycf1* gene were utilized to reconstruct a phylogenetic tree (Figure 6). Phylogenetic trees based on the entire plastid genome, protein-coding genes, and the *ycf1* gene displayed closely aligned topologies. However, in the phylogenetic trees derived from the whole plastomes (Figure 5) and protein-coding gene sequences (Supplementary Figure S1), the cluster containing the subgenus *Orithyia* with *T. sinkiangensis* was a close neighbor of the cluster of the subgenus *Eriostemones*. Conversely, in the ML tree based on the *ycf1* sequences, the subgenus *Orithyia* was closer to the subgenus of the *Tulipa* cluster (Figure 6).

## 4. Discussion

A comparative analysis was conducted on 14 complete plastome sequences of *Tulipa*, encompassing the two species examined in the present study and 12 additional samples sourced from GenBank. The assessment of the complete plastome sequences of *T. alberti* and *T. greigii* suggested genome sizes of 152,359 and 152,242 bp, respectively. These genome sizes aligned closely with the previously reported genome sizes of *T. buhseana* (152,062 bp) [34] and *T. patens* (152,050 bp) [36].

The plastid genomes of *T. alberti* and *T. greigii* are distinguished by their identical gene content and arrangements, comprising four distinct genomic parts [19,20,22]. The total GC contents of the plastomes of *T. alberti* (36.57%) and *T. greigii* (36.59%) were very similar, consistent with those observed in other angiosperm plastid genomes [23,24,28]. A GC content exceeding 40% was predominantly found within the IR region, compared to the LSC and SSC regions, as identified in a previous study on *Tulipa* species [39]. The elevated GC content observed in the IR region can be related to four ribosomal RNA genes [56,57].

The *Tulipa* plastomes analysis revealed 114 unique genes, including eighty protein-coding, thirty tRNA, and four rRNA genes (Table 2). Similar to most plant species, the *rps12* exhibits a trans-spliced nature, featuring duplicated 3′ ends within the inverted repeat region, whereas its 5′ end is situated in the LSC region [39]. However, variations in the number of protein-coding genes were also observed. As reported by Zhou et al. [33], in *T. altaica*, *psbJ*, and *rpl32* were absent from the annotation. Additionally, the pseudogenes *ycf1*, *rps19*, and two *ycf68*, identified in this study, were not annotated in certain published plastomes of *Tulipa* available in GenBank. The absence of the *infA* gene remained consistent across both examined plastid genomes, a recurring observation in other *Tulipa* plastomes [33,34,35,36,37,38,39]. These differences, including gene loss, may be caused by misannotation or gene transfer to the nucleus, as previously observed in *Populus* [58].

DNA microsatellites, or SSRs, are a type of DNA marker that are valued for their high polymorphism, which enables the accurate assessment of genetic diversity, population structure, and evolutionary relationships [59]. Their polymorphic nature, co-dominance, genome-wide distribution, and genotyping make them invaluable tools in genetic and molecular biology research [60]. In this study, 415 potential SSRs were detected; mononucleotide repeats were the most abundant (58.31% of the total SSRs), followed by dinucleotide, tetranucleotide, trinucleotide, and hexanucleotide repeats (Table 3). Nearly all mononucleotide repeats consisted of A/T (232), and 10 were composed of C/G. Among the dinucleotide repeats, AT/AT resulted in 95 repeats, whereas AG/CT accounted for 44. These findings agree with prior studies, indicating that microsatellites in plastid genomes normally consist of polyadenine or polythymine repeats [61,62]. Hexanucleotide repeats (1.2%) were extremely uncommon in both *Tulipa* plastomes, which is consistent with the previously reported results in *Xanthium sibiricum* [30]. These identified SSRs may be important for the assessment of the genetic diversity level in wild populations of *Tulipa* species. Utilizing the identified SSR markers may offer valuable information on the genetic variability within populations, aiding the development of efficient preservation strategies for *Tulipa* species.

Additionally, the variable regions identified within the plastid genomes are invaluable molecular markers, providing an essential platform for profiling studies and taxonomic analysis [28,63]. In the present study, *ycf1* was identified as having the highest variability, with a Pi value of 0.01859 (Figure 3), which is aligned with earlier reports [64,65]. Therefore, we suggest that the *ycf1* gene region, which displays relatively high sequence variability, could serve as an informative DNA marker and valuable resource for taxonomic studies of *Tulipa* species. This conclusion was supported by the ML phylogenetic tree of the studied *Tulipa* species based on *ycf1* (Figure 6), as the dendrogram nearly mimicked the trees based on the data of the whole plastomes (Figure 5) and protein-coding genes (Supplementary Figure S1).

The utilization of nucleotide sequences of plastome has gained popularity for studying relationships across various plant taxonomic levels, providing valuable insights into evolutionary studies owing to their conserved genome structure and relatively stable gene composition. Phylogenetic analyses have been performed on representatives of the family Liliaceae [29,64,66,67], including *Tulipa* species [39,68]. Despite several published reports on *Tulipa* phylogeny using genome size, ploidy level, and different DNA markers [2,5,7,8,10], the molecular taxonomy of this genus remains controversial [5]. This is also true for the subgenus *Tulipa*, for which Zonneveld suggested seven sections, including *Vinistriatae* [2]. In this study, 14 species of the *Tulipa* were analyzed using complete plastid genome sequences (Figure 5). The eight species in the subgenus *Tulipa* included four species from the section *Tulipa* and two species from the sections *Vinistriatae* and *Kolpakowskiana*, according to Zonneveld [2]. Assessment of the phylogenetic tree showed that *T. alberti* and *T. greigii* collected in Kazakhstan (both from the section *Vinistriatae*) clustered together in one subclade and formed a larger clade with species from the sections *Tulipa* (*T. gesneriana*) and *Kolpakowskianae* (*T. iliensis* and *T. altaica*) with a strong bootstrap value (Figure 5). Wilson [68] suggested the hypothesis of merging the sections *Vinistriatae* and *Kolpakowskianae*, along with the other three sections (*Tulipa*, *Spiranthera*, and *Lanatae*) into a single *Tulipa* section. The results of the phylogenetic analysis in the present work align with Wilson’s [68] suggestion. However, further research is necessary to confirm this proposed merger.

Therefore, this study confirms that the increasing availability of plastid genome sequences will further help reveal the evolutionary relationship of species in the genus *Tulipa*. The data from these two plastid genome sequences can be used as a reference for future plastome-based molecular taxonomic studies of the genus *Tulipa*.

## 5. Conclusions

This study presents a complete characterization of the plastid genomes of species *T. alberti* and *T. greigii* from the genus *Tulipa*. The highest genetic variability was recorded for *ycf1*, suggesting that this genetic factor could be recommended as one of the most informative DNA barcodes for discriminating *Tulipa* species. The assessment of the two plastid genomes provides a wealth of information for developing SSR markers for future evaluation of genetic diversity within the genus. Phylogenetic analysis using the complete plastid genome sequences of 14 *Tulipa* species, including *T. alberti* and *T. greigii*, indicated the high relatedness of species within the subgenera *Tulipa*. The generated phylogenetic tree results agree well with the hypothesis that species in the sections *Vinistriatae* and *Kolpakowskiana* and those in the section Tulipa should be combined into a single section, *Tulipa*. Still, additional studies are necessary to confirm this proposed merger.

## Figures and Tables

**Table 1:** Plastid genome characteristics of *Tulipa alberti* and *Tulipa greigii*.

	*Tulipa alberti*	*Tulipa greigii*
GenBank numbers	OR456441	PP338772
Genome size (bp)	152,359	152,242
LSC (bp)	82,367	82,256
SSC (bp)	17,248	17,244
IR (bp)	52,744	52,742
Number of total genes	136	136
Protein-coding genes	80	80
tRNAs	30	30
rRNAs	4	4
Total GC content (%)	36.57	36.59
LSC GC content (%)	34.49	35.53
SSC GC content (%)	29.85	29.88
IR GC content (%)	42.01	42.01

**Table 2:** List of genes annotated in plastomes of *T. alberti* and *T. greigii*.

Category	Group of Genes	Name of Genes
Self-replication	Transfer RNA	*trnA-UGC* ^a^ (×2), *trnC-GCA*, *trnD-GUC*, *trnE-UUC*, *trnF-GAA*, *trnfM-CAU*, *trnG-GCC* ^a^, *trnG-UCC*, *trnH-GUG* (×2), *trnI-CAU* (×2), *trnI-GAU* ^a^ (×2), *trnK-UUU* ^a^, *trnL-CAA* (×2), *trnL-UAA* ^a^, *trnL-UAG*, *trnM-CAU*, *trnN-GUU* (×2), *trnP-UGG*, *trnQ-UUG*, *trnR-ACG* (×2), *trnR-UCU*, *trnS-GCU*, *trnS-GGA*, *trnS-UGA*, *trnT-GGU*, *trnT-UGU*, *trnV-GAC* (×2), *trnV-UAC* ^a^, *trnW-CCA*, *trnY-GUA*
	Ribosomal RNA	*rrn4.5* (×2), *rrn5* (×2), *rrn16* (×2), *rrn23* (×2)
	RNA polymerase	*rpoA*, *rpoB*, *rpoC1* ^a^, *rpoC2*
	Small subunit of ribosome	*rps2*, *rps3*, *rps4*, *rps7* (×2), *rps8*, *rps11*, *rps12* ^a^ (×2), *rps14*, *rps15*, *rps16* ^a^, *rps18*, *rps19*, *rps19* ^c^
	Large subunit of ribosome	*rpl2* ^a^ (×2), *rpl14*, *rpl16* ^a^, *rpl20*, *rpl22*, *rpl23* (×2), *rpl32*, *rpl33*, *rpl36*
Genes for photosynthesis	NADH dehydrogenase	*ndhA* ^a^, *ndhB* ^a^ (×2), *ndhC*, *ndhD*, *ndhE*, *ndhF*, *ndhG*, *ndhH*, *ndhI*, *ndhJ*, *ndhK*
	Photosystem I	*psaA*, *psaB*, *psaC*, *psaI*, *psaJ*
	Photosystem II	*psbA*, *psbB*, *psbC*, *psbD*, *psbE*, *psbF*, *psbH*, *psbI*, *psbJ*, *psbK*, *psbL*, *psbM*, *psbN*, *psbT*, *psbZ*
	Subunits of cytochrome	*petA*, *petB* ^a^, *petD* ^a^, *petG*, *petL*, *petN*
	ATP synthase	*atpA*, *atpB*, *atpE*, *atpF* ^a^, *atpH*, *atpI*
	Rubisco	*rbcL*
Other genes	Maturase	*matK*
	Protease	*clpP* ^b^
	Envelope membrane protein	*cemA*
	Subunit of acetyl-CoA-carboxylase	*accD*
	C-type cytochrome synthesis gene	*ccsA*
Genes of unknown function	Hypothetical chloroplast reading frames	*ycf1*, *ycf1* ^c^, *ycf2* (×2), *ycf3* ^b^, *ycf4*, *ycf15* (×2), *ycf68* ^c^, *ycf68* ^c^

^a^ one intron-containing gene; ^b^ two intron-containing genes; ^c^ pseudo gene; (×2) duplicated genes.

**Table 3:** Characteristics of detected simple sequence repeats in the plastid genomes of *T. alberti* and *T. greigii*.

Type	Repeat Unit	*T. alberti*	*T. greigii*	Total	%
Mono-	A/T	116	116	232	58.31
	C/G	5	5	10	
Di-	AG/CT	22	22	44	33.49
	AT/AT	48	47	95	
Tri-	AAT/ATT	5	4	9	2.17
Tetra-	AAAG/CTTT	1	1	2	4.82
	AAAT/ATTT	6	6	12	
	AATG/ATTC	1	1	2	
	AATT/AATT	1	1	2	
	AGAT/ATCT	1	1	2	
Hexa-	AAAAAC/GTTTTT	2	2	4	1.20
	AGCCAT/ATGGCT	0	1	1	
Total		208	208	415	100

## Data Availability

Plastid genome data are available in the National Center for Biotechnology Information Database under the accession numbers OR456441 (*T. alberti*) and PP338772 (*T. greigii*).

## Supplementary Materials

The following supporting information can be downloaded at https://www.mdpi.com/article/10.3390/genes15111447/s1, Figure S1: Phylogenetic tree based on nucleotide sequences of protein-coding genes; Table S1: List of simple sequence repeats (SSRs) in *T. alberti* and *T. greigii* plastomes.
