# Supplementary figures and images for: Assessment of Complete Plastid Genome Sequences of Tulipa alberti Regel and Tulipa greigii Regel Species from Kazakhstan
Source: Genes (Basel). 2024 Nov 9;15(11):1447. doi: 10.3390/genes15111447 (PMC11593697; doi:10.3390/genes15111447)

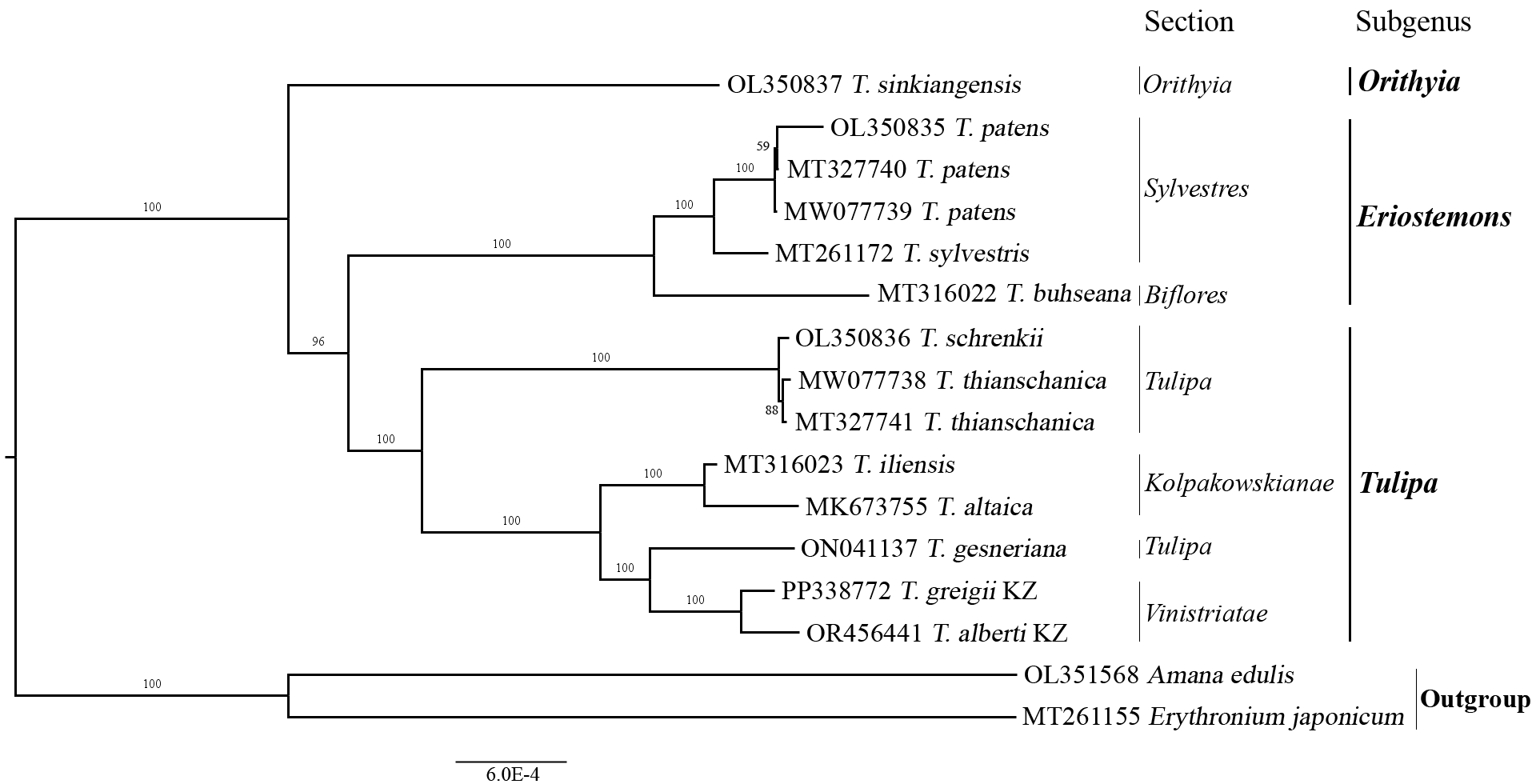

Supplement: Supplementary file 1 [file genes-15-01447-s001.zip › Figure S1 Phylogenetic tree based on nucleotide sequences of protein-coding genes.png]
